# Distribution of Dietary Phospholipids in Selected Agri-Foods: Versatile Nutraceutical Ingredients

**DOI:** 10.3390/foods13223603

**Published:** 2024-11-11

**Authors:** Ho-Chang Kim, Eun-Ju Cho, Hyeon-Jun Chang, Jung-Ah Shin, Jeung-Hee Lee

**Affiliations:** 1 Department of Food and Nutrition, Daegu University, Gyeongsan 38453, Republic of Korea; ghckd959@gmail.com (H.-C.K.); joeunju2001@gmail.com (E.-J.C.); chj931116@naver.com (H.-J.C.); 2 Department of Marine Bio Food Science, Gangneung-Wonju National University, Gangneung 25457, Republic of Korea; jashin@gwnu.ac.kr

**Keywords:** functional ingredient, phospholipid, method validation, agri-foods

## Abstract

Phospholipids (PLs) play a crucial role in the nutraceutical field due to their various health benefits, including supporting acetylcholine production, enhancing cell membrane fluidity, and promoting cognitive functions. This study aimed to investigate the PL composition of selected agri-foods, including grains, vegetables, and fruits, and assess the effects of cooking methods. The major PLs identified in most agri-foods were phosphatidylethanolamine (PE) and phosphatidylcholine (PC). Additionally, lyso-phosphatidylethanolamine and lyso-phosphatidylcholine were found in rice, grains, and wheat, while *N*-acyl-phosphatidylethanolamine was detected in grains, wheat, and some vegetables. Phosphatidylinositol was present in fruits and vegetables, and phosphatidylserine was exclusively found in mushrooms. The PL composition was influenced by cooking methods, with boiling, steaming, blanching, and roasting increasing the PL content, while salting tended to decrease it. Although most agri-foods contained higher levels of PC than PE, citrus fruits under long-term low-temperature storage had significantly more PE than PC. This study established a PL database for the selected agri- and processed/cooked foods, providing insights into changes in PL composition and content based on cooking methods. Given the important health functions of each PL, consuming various agri-foods and incorporating different cooking methods for optimal health benefits is advisable.

## 1. Introduction

Phospholipids (PLs) are one of the most important lipid categories in foods involved in multiple biological functions. They occur naturally as major components of the lipid bilayer of cell membranes. PLs are amphipathic molecules with hydrophobic fatty acid chains and hydrophilic moieties such as choline, serine, inositol, or ethanolamine, forming phosphatidylcholine (PC), phosphatidylserine (PS), phosphatidylinositol (PI), or phosphatidylethanolamine (PE), *N*-acyl-phosphatidylethanolamine (NAPE), lyso-phosphatidylethanolamine (LPE), and lyso-phosphatidylcholine (LPC) [1]. The content and composition of PLs in food are affected by both the variety of food and the cooking or processing methods. PC and PE are the major PLs in most foods; however, NAPE is found in wheat products, while sphingomyelin is present in animal-based foods [2]. Furthermore, in rice, boiling facilitates the extraction of lyso-PLs (LPLs), such as LPE and LPC, as starch gelatinizes [3]. High salinity activates phospholipase D (PLD), which degrades membrane PLs [4], and cold storage can lead to enzymatic reactions that modify PL composition [5].

Several roles of dietary PLs in health and disease have been reported since the early 1900s [6]. For instance, PC is involved in lipid digestion and absorption [7,8], acetylcholine production (a neurotransmitter involved in brain activity) [9], the prevention of arteriosclerosis and myocardial infarction by the reduction of blood cholesterol concentration [10,11], and exertion of an anti-inflammatory effect in arthritis [12]. PE increases cell membrane fluidity [13] and ameliorates non-alcoholic steatohepatitis by stimulating triglyceride and fatty acid metabolic regulators [14]. PS positively affects gut health [15]; improves learning, memory, and other cognitive functions; helps with negative mood (depression) improvement; and eases anxiety [16]. PE and PS are more abundant in the brain than in other tissues [17], and liver damage may occur when the PC/PE ratio decreases [18]. PI attenuates a liver function disorder caused by a high-fat diet in mice [19] and promotes hepatic uptake and biliary secretion of plasma cholesterol [20]. Among Lyso-PLs, LPE affects plant growth and development [21]. LPC has an inhibitory effect related to acetylcholine-mediated vasodilation [22], and with its unsaturated fatty acids, including C20:4, C20:5, or C22:6, it has demonstrated anti-inflammatory activity in zymosan A-induced peritonitis [23].

Choline is important for synthesizing acetylcholine and PLs in cell membranes, as well as for methyl group metabolism and cholinergic neurotransmission in humans [24]. Since 1998, the US Food and Nutrition Board of the Institute of Medicine has been recommending a choline intake of 425–550 mg/day as adequate intake (AI) [25]. The US Department of Agriculture has developed a choline database (DB) that enables researchers and consumers to estimate choline intake from the dietary intake of 630 common foods [26]. However, comprehensive information on individual dietary PLs in common foods remains limited. In Republic of Korea, the Rural Development Administration (RDA) has been building Meta DB, a database for functional components, including PLs, since 2022.

Interest in functional food ingredients is increasing not only in Republic of Korea but also globally, with the public increasingly seeking information regarding the functional components of the foods they consume daily. Particularly, as the important functionalities of individual dietary PLs in the human body are highlighted, the development of functional materials is actively ongoing in the pharmaceutical industry. However, information on the type and quantities of PLs contained in various agri-foods and the related changes in content after processing or cooking is insufficient. As agricultural foods are generally consumed in cooked forms, understanding the PL composition after cooking compared with that in raw foods is essential. The normal dietary intake of PLs is 2–8 g/day, representing 1–10% of total daily fat intake [27]. However, no established dietary intake recommendations exist for total or individual PLs. Therefore, building a foundational database by providing the PL profiles of both raw and cooked foods to establish dietary intake recommendations is imperative.

This study aims to investigate the PL composition in a selected variety of raw and processed/cooked agri-foods and evaluate the impact of cooking methods on PL composition. The findings are intended to provide foundational data for building a PL DB that could be valuable for the bio-health food industry and public consumers.

## 2. Materials and Methods

### 2.1. Chemicals and Reagents

As standard materials for PL analysis, NAPE, PE, PC, and PS were purchased from Sigma-Aldrich (St. Louis, MO, USA), and PI, LPE, and LPC were obtained from Avanti Polar Lipids, Inc. (Alabaster, AL, USA). Chloroform and methanol were purchased from Samchun Pure Chemicals (Seoul, Republic of Korea). Iso-propanol, *n*-hexane, and water were purchased from Fisher Scientific Korea Ltd. (Incheon, Republic of Korea), and trimethylamine was purchased from Sigma-Aldrich.

### 2.2. Sample Preparation

Grains, wheat flour, vegetables, and fruits, except for lime (imported from Mexico), were cultivated and harvested in Republic of Korea in 2023 and then collected for analysis. Their processed products, manufactured domestically, were purchased from a local Korean market; their photos and detailed information are listed in Table 1 and Table 2. Grain products, including rice (polished rice, five varieties; brown rice, two varieties), oats, buckwheat, barley, Job’s tear, and corn cereal, were purchased and cooked according to the methods described in Table 1. Wheat flour (three varieties) and products (six varieties) were purchased and cooked by boiling (Table 1). For the vegetables, soybean sprout, deodeok root, balloon flower (doraji), bracken, carrot, onion, cabbage, Korean cabbage, and mushroom were obtained and cooked by blanching, roasting, steaming, stir-frying, or boiling (Table 2). For the fruits, mandarin orange (six varieties), kumquat, lime, lemon, blueberry, pear, and apple, as well as juices and jams, were purchased. The raw and cooked/processed samples were freeze-dried and stored in powdered form at −20 °C (except the juices).

### 2.3. Lipid Extraction

A freeze-dried food sample (2 g), folch solution (24 mL, chloroform:methanol = 2:1, *v*/*v*), and distilled water (6 mL) were placed in a centrifuge tube and mixed for 2 min. After centrifugation for 10 min at 1763× *g* (Labogene 1248; Gyrozen Co., Ltd., Seoul, Republic of Korea), the lower layer was obtained. Extraction was repeated twice by adding 12 mL of chloroform and 1 mL of methanol, after which the lower layer was combined. After passing through a sodium sulfate column, the extracted lipid was concentrated and completely dried using N_2_.

### 2.4. Analysis of Phospholipids

Each PL was analyzed using a high-performance liquid chromatograph (HPLC, Shimadzu Corp., Kyoto, Japan) equipped with a LiChrospher100 Diol column (250 × 4 mm, 5 μm; Merck, Darmstadt, Germany) and an evaporative light scattering detector (ELSD; Sedex LT-ELSD Model 80LT, Sedere, France). The temperatures of the column oven and evaporation for ELSD were set at 40 and 50 °C, respectively. N_2_ was used as a nebulizing gas at a pressure of 2.5 bars. The extracted lipids were dissolved at a concentration of 1.0–4.0 mg/mL with *n*-hexane: iso-propanol (3:1, *v*/*v*). The injection volume was 20 µL, and the flow rate of the mobile phase was set at 1.0 mL/min. The mobile phases were solvent A (*n*-hexane: iso-propanol: acetic acid: triethylamine = 81.42:17.00:1.50:0.08, *v*/*v*/*v*/*v*) and solvent B (iso-propanol: water: acetic acid: triethylamine = 84.42:14.00:1.50:0.08, *v*/*v*/*v*/*v*) with the following gradient: 0–3 min (100% A), 3–8 min (95% A), 8–15 min (80% A), 15–25 min (60% A), 25–40 min (20% A), 40–45 min (50% A), 45–47 min 100% A), and 45–80 min (100% A). The calibration curve was obtained using individual PL standard solutions, and the PL content was calculated and expressed as mg/100 g of sample (dried). Each sample was analyzed in triplicate.

### 2.5. Method Validation

The analytical methods for PL qualification and quantification were validated based on specificity, linearity, sensitivity, accuracy, and precision according to the International Council for Harmonization (ICH) guidelines [28].

#### 2.5.1. Specificity and Linearity

The specificity of the PL analytical methods was validated using standards, where the peaks of individual PLs in the samples were identified based on the retention time of standards from chromatograms obtained by HPLC-ELSD. To validate linearity, standard solutions were prepared at concentrations of 4.46–1000 μg/mL for PC, 2.23–571.40 μg/mL for LPC, and 4.46–571.40 μg/mL for NAPE, PE, PS, PI, and LPE. Linear regression analysis was used to evaluate the linearity of calibration curves with the quadratic function, and the related linearities (coefficients of determination, *R*^2^) were obtained.

#### 2.5.2. Sensitivity and Precision

The sensitivity of the PL analytical methods was evaluated based on the limit of detection (LOD) and the limit of quantification (LOQ). The peak areas of six blanks from HPLC chromatograms were obtained, and the average area and standard deviation (SD) were calculated. The average area was multiplied by 3.3 and 10, and their respective concentrations (A and B) were obtained. The calibration curve was plotted with five concentrations, including A and B, and then the LOD (3.3× SD/S) and LOQ (10× SD/S) were calculated, where *S* is the slope of the calibration curve. Precision was assessed through repeatability, with precision-repeatability determined as the SD and relative SD (RSD, %) of triplicates. To ensure the precision of the analytical methods for major PLs, RSD should be ≤2%, and for low-level impurities, an RSD of 5–10% is usually acceptable.

### 2.6. Statistical Analysis

Analysis of variance was performed using SAS 9.4 (SAS Institute, Inc., Cary, NC, USA). Statistical differences were determined using Student’s *t*-test for two groups or Duncan’s multiple range test for three or more groups. Statistical significance was set at *p* < 0.05.

## 3. Results and Discussion

### 3.1. Method Validation for Phospholipid Analysis

**Figure 1:**
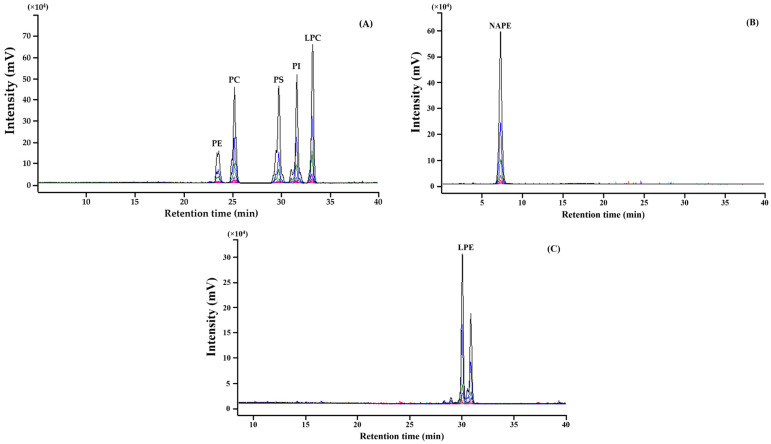
HPLC-ELSD chromatograms of phospholipid standard: (**A**) PE, PC, PS, PI, LPC, 2–571 μg/mL; (**B**) NAPE, 4~571 μg/mL; (**C**) LPE, 4~571 μg/mL (light green, 2 μg/mL; pink, 17 μg/mL; green, 142 μg/mL; blue, 285 μg/mL; black, 571 μg/mL).

**Figure 2:**
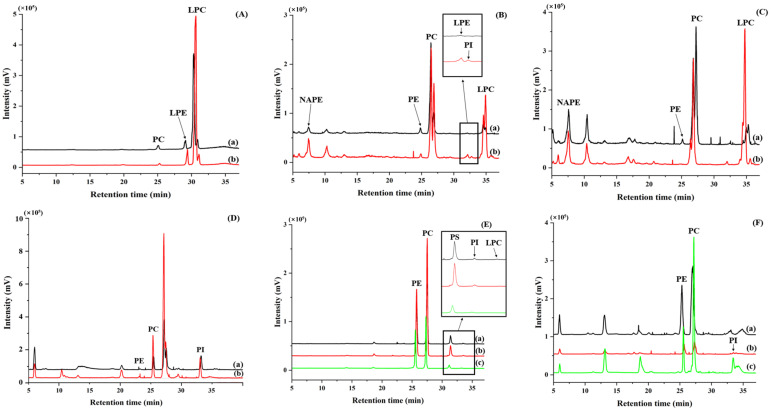
HPLC-ELSD chromatograms of phospholipids in common raw and processed foods. (**A**) (a) Raw polished odae rice, (b) cooked polished odae rice. (**B**) (a) Raw tetrastichum barley, (b) cooked tetrastichum barley. (**C**) (a) Korean wheat flour, (b) boiled spaghetti. (**D**) (a) Raw carrot, (b) steamed carrot. (**E**) (a) Raw king oyster mushroom, (b) boiled king oyster mushroom, (c) roasted king oyster mushroom. (**F**) (a) Redhyang, (b) lemon, (c) busa apple.

The HPLC-ELSD analytical method for PL quantification was validated for specificity, linearity, and sensitivity, as presented in Figure 1 and Figure 2 and Table 3. For specificity, the standards of seven PLs were separated and identified in HPLC chromatograms (Figure 1). Linearity was evaluated using a calibration curve and coefficient of determination (*R*^2^) for each PL standard. The calibration curve was expressed using a quadratic function, and its *R*^2^ values were >0.99 (Table 3). The individual PLs in raw and cooked/processed agri-foods (grains, vegetables, and fruits) were qualified based on their retention times in the HPLC chromatogram and quantified using the calibration curve (Figure 2).

The sensitivity of the PL analysis was expressed in terms of LOD and LOQ of the HPLC-ELSD method (Table 2). The LOD ranged from 0.40 to 2.62 μg/mL and was lowest for LPC, followed by PC, NAPE, PS, LPE, PI, and PE, showing satisfactory limits. The LOQ ranged from 1.03 to 9.67 μg/mL and was lowest for LPC, followed by PC, NAPE, PI, LPE, PE, and PS. The LOD value for the quantitative analysis of PLs using HPLC-ELSD was lower than that of a previous study [29]. The present study focused on quantitatively analyzing agri-food with relatively low PL content, relying on the analytical equipment and methods. Therefore, the low LOD and LOQ values suggest the potential for precise analysis and quantification of PLs in common raw and processed agri-foods, even at low levels.

### 3.2. Phospholipid Composition of Rice

The lipid contents of polished and brown rice were 0.77–1.74 and 2.94–3.95%, respectively, and the total PL content was 67.21–117.21 and 145.98–174.14 mg/100g, respectively. Particularly, brown rice exhibited higher lipid and PL contents compared with those of polished rice (Figure 2A and Table 4). Among polished rice varieties (five varieties), the major PLs identified included PC, LPE, and LPC, with LPC being the most abundant, and PE was either present in trace amounts or not detected. In contrast, brown rice (two varieties) contained PE, PC, LPE, and LPC as major PLs. In the same varieties (‘odae’ and ‘saeilimi’), brown rice exhibited significantly higher levels of PE, PC, and LPE (*p* < 0.05), whereas LPC content was significantly lower (*p* < 0.05) than in polished rice. In ‘odae’ and ‘saeilimi’ brown rice, the total PL content was 1.47- and 1.29-fold higher, respectively; this is largely attributed to significantly higher levels of PC (12.7- and 6.6-fold higher, respectively) and LPE (1.7- and 1.5-fold higher, respectively). This indicates that brown rice contained higher lipid and PL concentrations than those in polished rice, with considerable differences in the composition and abundance of specific PL species across rice varieties.

Typically, rice is cooked through soaking and boiling before being consumed, and the cooking method reduces the originally contained lipid content by 25.0–42.6% in polished rice and 12.6–19.2% in brown rice (Table 4). After cooking, the PC and PE contents of rice were significantly decreased (*p* < 0.05), and the LPE and LPC contents of brown rice significantly increased 1.45–1.57- and 1.45–1.67-fold, respectively (*p* < 0.05); otherwise, these contents in polished rice showed a tendency to either increase or decrease. Rice grain comprises bran, endosperm, and germ; lipids are primarily concentrated in bran and germ fractions. Brown rice is a whole-grain rice, whereas polished rice has the bran and germ removed. This is why brown rice contains higher lipid amounts than those in polished rice. Rice grain mainly consists of starch (amylose and amylopectin) and a much smaller amount of lipids. PLs constituted 3.9–15.2 and 4.4–5.0% of the lipid content of polished and brown rice, respectively. After cooking, the PL concentration increased to 10.5–16.4% and 5.8–6.0%, respectively (Table 4). In rice, PLs in the bran and germ comprise a single layer, whereas lipids in the endosperm form an amylose-lipid complex [3]. As internal starch lipids, LPLs are effectively extracted under rigorous conditions (i.e., high temperature). Specifically, because cooked rice undergoes a boiling process at high temperatures, resulting in starch gelatinization, extracting LPLs becomes easier. Therefore, the content of LPC and LPE appeared to be higher in cooked rice compared with that in raw rice.

Choline, the hydrophilic moiety that forms PC and LPC, the major PLs of rice, is important for synthesizing acetylcholine and cholinergic neurotransmission in humans, promoting brain activity [24]. Brown rice contains not only PC and LPC but also PE and LPE; therefore, consuming brown rather than polished rice is more beneficial for health. Rice is the single most important staple food in the world, and the cooked and processed forms make it an important dietary source of PLs impacting human health globally.

### 3.3. Phospholipid Composition of Other Grains

The lipid and PL contents of oats, buckwheat, barley, and Job’s tears, which are domestic grains, were analyzed along with their processed products before and after cooking. Lipid content ranged from 3.92% in buckwheat to 10.30% in oats. Oats, buckwheat, and barley showed a decrease in lipid content after cooking (steaming or boiling), whereas Job’s tears showed an increase in lipid content after steaming (Table 5). PL content was 6.85–10.78% of lipid content in the grains, and the total PL content was highest in oats, followed by barley, buckwheat, and Job’s tear, in decreasing order (Table 5). PC was the most abundant PL, followed by PE, LPC, LPE, NAPE, and PI. Unlike in rice, NAPE and PI were detected in these grains [2] (Figure 2B and Table 5). Barley, oats, and rye flour reportedly contain NAPE at 0.20–0.25 mg/g of dry weight, consistent with the findings of the current study [2]. The total PL content decreased after cooking for oats (*p* > 0.05) and barley (*p* < 0.05) but increased for Job’s tear (*p* < 0.05). Although PL, PE, PC, and LPE levels decreased post-cooking, LPC content significantly increased (*p* < 0.05). Oats and barley exhibited a 12.9 and 15–27% reduction in total PL content, respectively. Conversely, LPC content increased by 21.0, 62.7–139.3, and 119.9% in oats, barley, and Job’s tear, respectively, after cooking. Buckwheat noodles showed a decrease in PC content after boiling and increased LPE and LPC content, resulting in an 18.41% increase in total PL content. The increase in the LPC content of grain after cooking follows a pattern similar to that of cooked rice. This suggests that as grains cook and starch gelatinizes through boiling or steaming at high temperatures, the extraction of LPC—embedded as internal starch lipids—becomes easier, leading to higher LPC contents. Oatmeal contained NAPE, PE, PC, LPE, and LPC, whereas corn cereals contained only PC and LPC.

Oat, buckwheat, barley, and wheat are also major staple grains along with rice, but they contain NAPE and PI, unlike rice. NAPE occurs in relatively minor amounts in several different plants and is also found in a very small amount in mammalian tissues (e.g., the brain). NAPE is synthesized from fatty acids and PE in response to cell injury under certain stress conditions and is recognized as being cytoprotective, especially in the brain [30]. PI plays an important role in maintaining the normal physiological functions of the central nervous system [31] and promotes hepatic uptake and biliary secretion of plasma cholesterol [20]. As a core constituent of PI, inositol benefits patients with medical conditions such as insulin resistance and non-alcoholic fatty liver disease. Additionally, increased inositol intake is suggested to ameliorate several disorders, such as polycystic ovary syndrome, gestational diabetes, and poor sperm development [32]. Although PI is present in small amounts in buckwheat and barley, these grains also contain various PLs, including NAPE. Consequently, when consumed as staple grains alongside rice, they may help improve various disorders through the effects of individual PLs.

### 3.4. Phospholipid Composition of Wheat Flour and Its Products

Wheat flour (Korean and imported) and pan-frying powder (Korean) contained 1.78–2.66% lipids and major PLs such as PC, NAPE, and LPC, along with trace amounts of PE, resulting in a total PL content of 352.1–423.08 mg/100g (Table 6). Schaffarczyk et al. [33] reported that the PLs in wheat flour comprise NAPE, PC, and LPC, with NAPE present at 779 mg/kg of dry weight. Wheat flour is used in various foods and processed into different food products for global consumption, major products of which include Korean ramen, udon, plain, and chopped noodles and spaghetti (Table 6). Most wheat flour-based processed foods are sold in dry form and contain a very small amount of lipids (0.57–4.05%). Of these, PC has the highest content in wheat flour products in the present study, followed by LPC and NAPE. LPC content increased after cooking, resulting in an increase in total PL content.

Korean ramen exports from January to October 2023 amounted to $785.25 million, a 24.7% increase compared with the same period in 2022 [34]. Ramen is exported to about two-thirds of the countries worldwide, and export revenue is highest in China, the USA, and Japan, in that order. Ramen noodles are normally dehydrated by final deep-frying using palm oil before packaging for long storage [35]. Major palm oil constituents include triacylglycerols (over 95%) and remarkably lower levels of PLs (20–80 ppm) for oxidative stability and bleaching properties. This is why ramen noodles contained the highest lipid content (16.05%) among the noodles. After boiling for consumption, the lipid and total PL contents decreased by 15.5 and 6.6%, respectively (Table 6). Spaghetti (pasta) is among the most common and popular staple foods, and 14.3 million tons are reportedly produced annually worldwide, the top producer being Italy, followed by the USA and Brazil [36]. Spaghetti is made from durum wheat (*Triticum durum* Desf.). The WHO considers it a healthy, sustainable, and quality food model because of its nutritional profile, which includes low lipid and high protein content, readily digestible carbohydrates, and healthy components such as fiber, as well as its low-cost production and long shelf life. The final step in manufacturing spaghetti involves a drying process before distribution for sale. In the present study, dried spaghetti was boiled, resulting in a PC decrease of 19.9% and an LPC increase of 67.8%, consequently increasing total PLs by 8.9% (Table 5). Similar to rice, wheat is one of the most important cereal crops worldwide and is normally transformed into flour to produce a wide variety of staple foods in the human diet. Noodles, as wheat flour products, contained higher amounts of PLs, specifically LPC, PC, and NAPE, after cooking. In contrast, cooked rice contained PE and LPE, which were not identified in wheat flour products (Table 4 and Table 6). Therefore, a balanced staple diet that includes both rice and wheat is recommended.

### 3.5. Phospholipid Composition of Vegetables

The vegetables contained a small amount of lipids, ranging from 2.91 to 5.47% (excluding soybean sprouts, which contained 19.05%). The PL composition included NAPE, PE, PC, PI, and LPC. Although PE, PC, and PI were present in all vegetables, NAPE was detected only in deodeok, balloon flower root, and bracken, whereas LPC was found exclusively in mushrooms. Additionally, PL content increased after cooking (Table 7). The cooking methods included blanching, roasting, boiling, steaming, and stir-frying. The heating process induces alterations in the microstructure of the plant tissues, leading to tissue softening, thermal degradation of middle lamella pectins and other cell wall polysaccharides, and starch gelatinization. This resulted in easier PL extraction in the vegetables, leading to an apparently higher PL content after cooking [37].

Regarding the PL content of soybean sprouts, PC was the most abundant, followed by PE and PI. The PE and PC contents of soybean sprouts increased 3.95- and 4.35-fold, respectively, after blanching (*p* < 0.05). Soybean sprouts are culinary vegetables acquired by sprouting soybeans [38]. Generally, PC is the most abundant PL in soybeans, followed by PE and PI [39]. Deodeok and balloon flower root are traditional vegetables consumed in the Republic of Korea and East Asia and are also used for medicinal purposes. Their major PLs include PE, PC, and PI, with trace amounts of NAPE, whose contents were significantly increased by cooking (*p* < 0.05), with a greater increase observed from blanching rather than roasting. Bracken is commonly stored after drying, either directly or after boiling. Before consumption, it is rehydrated or boiled for use in cooking. Boiled/dried and boiled/dried/boiled bracken contained significantly higher levels of NAPE, PE, PC, and PI than those in the other vegetables (*p* < 0.05). Notably, when the boiled and dried bracken was boiled again, the content of all PLs increased significantly by 1.28- to 1.54-fold (*p* < 0.05).

PC, PE, and PI were the major PLs in carrots, cabbages, Korean cabbages, and onions. The PL content increased in these vegetables after cooking. In carrots, blanching resulted in the highest PL content, with 1.97- and 1.96-fold increases in PE and PC, respectively, compared with that in raw carrots (*p* < 0.05). Processed carrot juice contained small amounts of PE, PC, PI, and LPC. For cabbages, both PE and PC contents increased after boiling, whereas salted cabbages exhibited a 70% reduction in PC content compared with that in raw cabbages, leading to a 19% reduction in total PL content. High salinity in vegetables leads to salt stress on the cell membrane, which degrades due to PLD activation. In the PLD pathway, phosphatidic acid (PA) is produced by the hydrolysis of membrane PLs, such as PC and PE. The salt-induced accumulation of PA leads to a loss of cell membrane integrity, and the generated PA can be further broken down by phosphatases, acylhydrolases, and lipoxygenases into soluble molecules [4]. This salt stress likely contributed to the reduced PL content observed in cabbages. In many East Asian countries, including the Republic of Korea, most cabbages are prepared for kimchi at home or in factories following a salting process [40].

Shiitake mushrooms, widely consumed in the Republic of Korea, are dried immediately after harvesting for distribution, whereas king oyster mushrooms are distributed and consumed fresh. These mushrooms contained small amounts of fat (2.78–4.12%), with PLs comprising 49.2–55.6% of the total lipid content. The most abundant PLs were PE, PC, and PS, whereas PI and LPC were available in trace amounts (Table 7). The PL composition of mushrooms comprised PC, PE, PI, and PS, similar to the findings of [41]. Agri-foods such as rice, wheat, vegetables, and mushrooms generally contain higher levels of PC than those of PE. However, king oyster mushrooms had 1.5-fold more PE than PC and a significantly higher total PL content (1.67-fold) compared with that of shiitake mushrooms (*p* < 0.05). Boiling or roasting king oyster mushrooms tended to increase PE and PI contents, with boiling leading to a greater increase in PL content than roasting. Boiling or roasting king oyster mushrooms tended to increase PE and PI content, with boiling resulting in a greater overall increase in PL content than roasting. Although the PL composition of mushrooms varies by species, *Boletus* mushrooms (*Basidiomycota*) generally contain PC, PE, PS, and PI, with PC being the most abundant—approximately 7-fold higher than PE [42]. Mushrooms had an especially high PS content, ranging from 244.75 to 273.1 mg/100 g. Notably, PS was not detected in grains such as rice and wheat, vegetables, or citrus fruits. PS helps with human cognitive functions, including short-term memory formation, long-term memory consolidation, and the ability to create new memories [43]. Therefore, regular consumption of mushrooms with high PS content may improve cognitive functions.

### 3.6. Phospholipid Composition of Fruits

The PL composition of the Republic of Korea’s representative fruits, including citrus fruit, apple, pear, and blueberry, varied depending on the varieties (Table 8) [44,45]. The major PLs of citrus fruits were PE (123–226 mg/100g) and PC (96–143 mg/100g), with PI in trace amounts (9–26 mg/100g). According to previous reports, citrus fruits, including oranges, grapefruits, lemons, and limes, contain PLs, especially PE and PC, along with trace amounts of PI and PS, with PS being present in the lowest quantities [46,47,48]. The major PLs in pears and apples were PC, PE, and PI. The PL content in pears differed significantly between the two tested varieties (*p* < 0.05). PE and PC were the major PLs found in blueberries, which had the lowest total PL content among the analyzed samples. PC is generally the most abundant PL in food, and in the present study, it was also the most abundant in grains, wheat, vegetables, and fruits, followed by PE. However, citrus fruits exhibited an unusual pattern, with significantly higher PE content than PC (*p* < 0.05). For ‘satsuma,’ the early-maturing cultivar showed higher lipid content compared with that of the regular cultivar, with significantly elevated PE and PC levels (*p* < 0.05). This increase in PL content may be attributed to cell membrane degradation as the fruit ripens [49].

Low-temperature storage is the main postharvest technique used to preserve the quality and market value of fruits and vegetables [50]. This can drive changes in PLs due to cold stress. According to Wang et al. [5], the PC content of cold-stored blueberries was remarkably lower than that of non-refrigerated blueberries, whereas the PE content of blueberries cold-stored for 60 days increased significantly. In the plants, cold storage increases the activity of PLD, which catalyzes two reactions: hydrolysis of the diester bond of PLs producing PA and a hydrophilic headgroup, and transphosphatidylation of PL with alcohol forming a new phosphatidyl alcohol. Because PLD1 and PLD2—PLD isoforms are highly selective for PC, low storage temperature can activate PC hydrolysis, resulting in PA and choline, thus reducing PC content [51]. For the transphosphatidylation of PC, PLD showed a preference for ethanolamine [52]. Long-term low-temperature storage may have activated PLD in citrus fruits such as oranges, leading to hydrolysis and subsequent transphosphatidylation of PC to PE, resulting in the relatively increased PE content. Additionally, PA and choline generated from PC hydrolysis are likely present in citrus fruits.

PA is a lipidic mediator involved in many cell signaling routes, and choline is a precursor of the neurotransmitter acetylcholine. Because de novo synthesis of choline alone is insufficient to meet human requirements, choline was officially recognized as an essential nutrient by the Institute of Medicine in 1998 [25]. The AI of choline for adult women and men is 425 and 550 mg/day, respectively. The recommended AI of choline for pregnant and lactating women is 450 and 550 mg/day, respectively, as the demand for choline is especially high and supplementation is critical in these physiological conditions [53].

## 4. Conclusions

This study provided a comprehensive analysis of PL composition in selected agricultural foods and demonstrated that PL content was affected by cooking methods. The primary PLs identified—NAPE, PE, PC, PS, PI, LPC, and LPE—varied in content across foods, with PE and PC predominantly present in all samples. Notably, NAPE was detected in grains, wheat, and some vegetables; PI was found in buckwheat, barley, vegetables, and fruits; LPC was present in rice, grains, flour products, and mushrooms; LPE was found in rice and grains; and PS was exclusively found in mushrooms. Cooking methods, particularly steaming, boiling, and blanching, notably affected PL content, with LPC content generally increasing post-cooking, suggesting the potential role of cooking in enhancing specific PL availability. As agricultural foods are mostly consumed in cooked forms, analyzing PLs in these cooked states offers relevant insights into their nutritional impact. Whole grains, such as brown rice, can provide a higher PL intake. Consuming various food groups provides more comprehensive benefits from different PL types. To optimize PL retention and functionality, heat-based cooking methods are advantageous over high-salinity methods, which may reduce PL content.

This study established a foundational PL composition database for agricultural foods and underscored specific dietary and cooking recommendations to increase PL intake. Future research should expand PL profiles across a broader range of foods and further investigate the impact of various cooking and preservation methods.

## Figures and Tables

**Table 1:** Selected grain and wheat products for phospholipid analysis.

Samples	Scientific Name	Varieties				Samples	Scientific Name	Varieties			
**Grain products**											
Rice	*Oryza sativa*	Polished		Brown		Barley	*Hordeum vulgare* L.	Tetrastuchum, polished barley			
		Odae		Odae				Raw 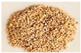		Boiled/Steamed 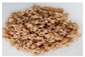	
		Raw 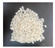	Boiled/Steamed 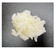	Raw 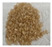	Boiled/Steamed 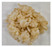						
		Saeilmi		Saeilmi			*Hordeum vulgare* var. *nudum Spenn*	Glutinous polished barley			
		Raw 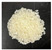	Boiled/Steamed 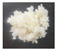	Raw 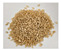	Boiled/Steamed 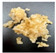			Raw 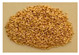		Boiled/Steamed 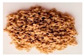	
Buckwheat	*Fagopyrum esculentum*	Grain (Peeled)	Polished powder	Noodle		Oat	*Avena nuda* L.	Polished		Oatmeal	
				Dried	Boiled			Raw 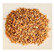	Boiled/Steamed 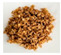	NA ^(1)^	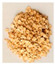
		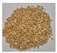	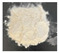	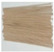	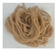						
Job’s tear	*Coix lacryma-jobi*	Grain (Polished)				Corn	*Zea mays*	NA		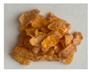	
		Raw 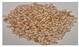		Boiled/Steamed 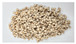							
**Wheat products**											
Wheat flour	*Triticum aestivum*	Pan frying powder				Korean wheat flour		All-purpose wheat flour			
		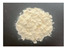				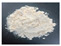		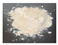			
Wheat flour products	*Triticum aestivum*	Ramen noodle				Wheat flour products	*Triticum aestivum*	Plain noodle			
		Dried 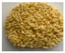		Boiled 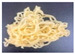				Dried 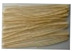		Boiled 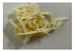	
		Udon						Jjolmyeon			
		Dried 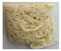		Boiled 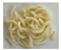				Dried 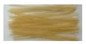		Boiled 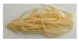	
		Chopped noodle					*Triticum durum*	Spaghetti			
		Dried 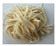		Boiled 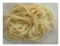				Dried 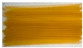		Boiled 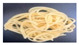	

^(1)^ Not available.

**Table 2:** Selected vegetables and fruits for phospholipid analysis.

Samples	Scientific Name	Varieties							Samples	Scientific Name	Varieties							
**Vegetables**																		
Soybean sprouts	*Glycine max* merr	Raw	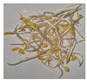		Blanched			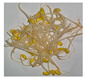	Cabbage	*Brassica oleracea* var. *capitata*	Raw 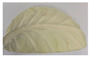			Blanched 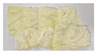		Steamed 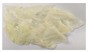		
Deodeok	*Codonopsis lanceolata*	Raw 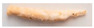		Blanched 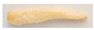		Roasted 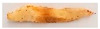			Ballon flower, root	*Platycodon grandiflorum*	Raw 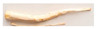			Blanched 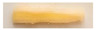		Roasted 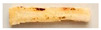		
Onion	*Allium cepa*	Raw 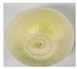		Blanched 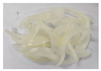		Roasted 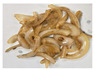			Carrot	*Daucus carota*	Raw		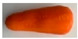		Blanched		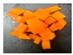	
											Steamed	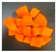		Stir-fried	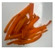	Juice		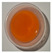
Bracken	*Pteridium aquilinum*	Boiled and Dried	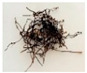		Boiled and Dried/Boiled			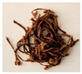	Shiitake mushroom	*Lentinus edodes*	Dried/Raw		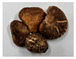		Dried/Boiled		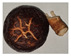	
Korean cabbage	*Brassica rapa* subsp. *pekinensis*	Raw 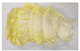		Boiled 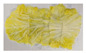		Salted 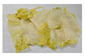			King oyster mushroom	*Pleurotus eryngii*	Raw 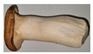			Boiled 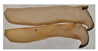		Roasted 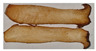		
**Fruits**																		
Citrus fruit	*Citrus hybrid*	Redhyang 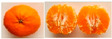			Hwanggeumhyang 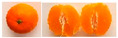				Citrus fruit	*Citrus reticulata*	Kumquat				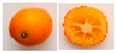			
	*Fortunella japonica* var. *margarita*	Cheonhyehyang 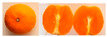			Hallabong 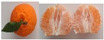					*Citrus aurantifolia* (Christm) *Swingle*	Lime				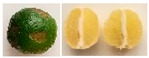			
	*Citrus unshiu*	Satsuma 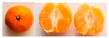			Satsuma Early-maturing cultivar 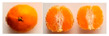					*Citrus limon*	Lemon				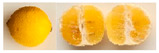			
Blueberry	*Vaccinium sect. Cyanococcus*	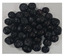							Apple	*Malus domesica Borkh*	Busa (peel removed)		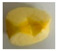		Busa (peel present)		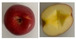	
Pear	*Pyrus pyrifolia*	Singo 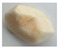		Won hwang 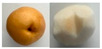			Pear juice 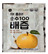				Apple juice		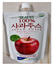		Apple jam		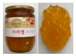	

**Table 3:** Method validation for phospholipid analyses with HPLC-ELSD.

Phospholipid	Linearity			Sensitivity	
	Calibration Curve ^(1)^	Calibration Range (μg/mL)	Coefficient of Determination (*R*^2^)	LOD ^(2)^ (μg/mL)	LOQ ^(3)^ (μg/mL)
NAPE	y = 21.356x^2^ + 11,958x − 108,340	4.46–571.40	0.9997	0.55	1.69
PE	y = 5.0965x^2^ + 3447.7x + 8131.3	4.46–571.40	0.9987	2.62	8.09
PC	y = 2.6891x^2^ + 13,202x − 168,970	4.46–1000.00	0.9933	0.42	1.22
PS	y = 25.466x^2^ + 3008.5x + 36,016	4.46–571.40	0.9985	1.98	9.67
PI	y = 10.667x^2^ + 10,968x − 159,382	4.46–571.40	0.9986	2.58	4.68
LPE	y = 2.9x^2^ + 10,165x − 133,177	4.46–571.40	0.9902	2.22	6.06
LPC	y = 5.5155x^2^ + 15,752x − 164,397	2.23–571.40	0.9977	0.40	1.03

^(1)^ y = ax^2^ + bx + c; y and x indicate peak area (mV) and concentration (μg/mL), respectively. ^(2)^ LOD: limit of detection. ^(3)^ LOQ: limit of quantification. NAPE, *N*-acyl-phosphatidylethanolamine; PE, phosphatidylethanolamine; PC, phosphatidylcholine; PS, phosphatidylserine; PI, phosphatidylinositol; LPE, lyso-phosphatidylethanolamine; LPC, lyso-phosphatidylcholine.

**Table 4:** Phospholipid composition of rice.

Korean Rice		Lipid Content (%)	Phospholipid Content (mg/100g Freeze-Dried)				
			PE	PC	LPE	LPC	Total PLs
**Polished rice**							
Dodam	Raw	1.74 ± 0.08 ^(1),^*^,(2),c,(3)^	- ^(4)^	8.51 ± 0.63 *^,c^	12.07 ± 1.64 ^e^	46.96 ± 5.02 ^c^	67.55 ± 7.27 ^d^
	Boiled/Steamed ^(6)^	1.12 ± 0.06 ^C,(5)^	-	6.93 ± 0.14 ^C^	16.02 ± 0.63 *^,C^	94.15 ± 2.62 *^,A^	117.11 ± 2.70 *^,C^
Headamsil	Raw	1.01 ± 0.04 *^,d^	3.45 ± 0.70 ^b^	12.48 ± 1.29 *^,c^	15.89 ± 1.76 ^c^	87.21 ± 7.87 ^a^	119.04 ± 11.60 *^,c^
	Boiled/Steamed	0.58 ± 0.08 ^E^	-	4.70 ± 0.49 ^C^	13.43 ± 1.60 ^CD^	76.48 ± 7.98 ^ABC^	94.60 ± 9.73 ^C^
Odae	Raw	0.92 ± 0.03 *^,d^	-	6.74 ± 0.79 *^,c^	12.34 ± 1.52 ^de^	65.98 ± 7.57 ^b^	85.06 ± 9.74 ^d^
	Boiled/Steamed	0.69 ± 0.05 ^D^	-	4.84 ± 0.29 ^C^	15.08 ± 1.08 ^CD^	90.52 ± 11.53 *^,AB^	110.44 ± 12.85 ^C^
Saeilmi	Raw	0.77 ± 0.12 *^,e^	-	8.42 ± 0.39 *^,c^	14.98 ± 1.18 *^,cd^	93.68 ± 6.99 *^,a^	117.08 ± 8.34 *^,c^
	Boiled/Steamed	0.52 ± 0.06 ^E^	-	3.51 ± 0.39 ^C^	10.59 ± 1.32 ^D^	70.92 ± 10.37 ^C^	85.03 ± 12.01 ^C^
Shindongjjin	Raw	0.97 ± 0.08 *^,d^	4.54 ± 0.41 ^b^	11.07 ± 0.66 *^,c^	13.47 ± 0.96 *^,cde^	83.46 ± 4.72 ^a^	112.54 ± 5.91 *^,c^
	Boiled/Steamed	0.56 ± 0.01 ^E^	-	3.57 ± 0.10 ^C^	11.30 ± 0.26 ^CD^	76.17 ± 2.80 ^ABC^	91.05 ± 2.93 ^C^
**Brown rice**							
Odae	Raw	3.95 ± 0.01 *^,a^	23.17 ± 3.10 ^a^	86.06 ± 11.45 ^a^	23.26 ± 0.70 ^a^	42.12 ± 4.97 ^c^	174.60 ± 19.67 ^a^
	Boiled/Steamed	3.19 ± 0.02 ^A^	16.15 ± 5.24 ^A^	64.39 ± 12.39 ^A^	35.49 ± 6.25 *^,A^	70.51 ± 14.32 *^,C^	186.54 ± 37.11 ^A^
Saeilmi	Raw	2.94 ± 0.08 *^,b^	19.81 ± 7.63 ^a^	53.68 ± 9.32 ^b^	19.92 ± 2.11 ^b^	52.85 ± 7.43 ^c^	146.26 ± 26.28 ^b^
	Boiled/Steamed	2.57 ± 0.03 ^B^	11.83 ± 0.64 ^A^	37.86 ± 3.98 ^B^	28.96 ± 2.04 *^,B^	74.60 ± 12.03 ^BC^	153.25 ± 18.31 ^B^

^(1)^ Mean ± SD. (*n* = 3). ^(2)^ * Means significantly differ between raw and cooked within the same polished or brown rice varieties using Student’s *t*-test (*p* < 0.05). ^(3) a–e^ Means with different lowercase letters in the same column significantly differ among raw polished and brown rice using Duncan’s multiple range test (*p* < 0.05). ^(4)^ - Not detected. ^(5) A–E^ Means with different uppercase letters in the same column significantly differ among cooked polished and brown rice using Duncan’s multiple range test (*p* < 0.05). ^(6)^ 30 min for polished rice; 40 min for brown rice.

**Table 5:** Phospholipid composition of grains.

Grains		Lipid Contents (%)	Phospholipids (mg/100 g of Freeze-Dried)						
			NAPE	PE	PC	PI	LPE	LPC	Total PL
**Oat**									
Grain (polished)	Raw	10.30 ± 0.07 ^(1),^*^,(2),a,(3)^	70.01 ± 21.11 ^a^	186.95 ± 57.19 ^a^	305.81 ± 69.93 ^ab^	- ^(4)^	57.99 ± 10.62 ^a^	84.86 ± 17.25 ^a^	705.63 ± 175.48 ^a^
	Boiled/Steamed ^(6)^	9.63 ± 0.10 ^A,(5)^	62.96 ± 16.46 ^A^	188.38 ± 53.72 ^A^	266.38 ± 50.41 ^A^	-	54.53 ± 11.68 ^A^	102.69 ± 22.88 ^B^	674.94 ± 154.37 ^A^
Oatmeal		6.83 ± 0.06	98.03 ± 23.05	200.37 ± 28.99	383.48 ± 60.88	-	47.69 ± 10.88	70.44 ± 14.69	800.01 ± 14.69
**Buckwheat**									
Grain (peeled)	Raw	3.92 ± 0.58 ^e^	-	85.82 ± 2.71 ^b^	263.29 ± 8.24 ^b^	39.77 ± 3.91 ^a^	-	-	388.88 ± 14.19
Polished powder	Raw	2.71 ± 0.01 ^f^	11.18 ± 0.33 ^c^	36.62 ± 4.34 ^d^	95.88 ± 2.95 ^d^	28.89 ± 1.10 ^b^	-	-	172.56 ± 7.09
Noodle	Dried	1.68 ± 0.03 ^g^	42.06 ± 1.36 ^b^	-	140.66 ± 1.23 *^,(5),d^	-	9.43 ± 0.50 ^c^	69.09 ± 2.18 ^b^	261.24 ± 4.38 ^c^
	Boiled	1.77 ± 0.02 *^,E^	42.41 ± 0.53 ^B^	-	115.18 ± 4.31 ^C^	-	13.38 ± 1.11 *^,B^	138.37 ± 9.80 *^,A^	309.34 ± 15.12 *^,C^
**Barley**									
Tetrastichum (polished)	Raw	4.48 ± 0.04 *^,d^	40.05 ± 0.93 ^b^	59.28 ± 2.83 *^,c^	313.96 ± 3.06 *^,a^	-	20.93 ± 0.19 *^,b^	48.93 ± 1.41 ^c^	483.14 ± 7.25 *^,b^
	Boiled/Steamed	2.25 ± 0.01 ^D^	42.89 ± 1.48 *^,AB^	17.57 ± 0.66 ^C^	229.13 ± 6.25 ^A^	10.79 ± 0.61 ^B^	16.00 ± 0.34 ^B^	117.11 ± 0.28 *^,B^	433.46 ± 7.20 ^B^
Glutinous (polished)	Raw	4.96 ± 0.11 *^,c^	30.34 ± 0.41 ^b^	58.19 ± 3.82 *^,c^	214.28 ± 9.51 *^,c^	20.60 ± 1.04 *^,c^	23.85 ± 1.07 *^,b^	95.39 ± 7.25 ^a^	442.63 ± 20.49 ^b^
	Boiled/Steamed	3.00 ± 0.03 ^C^	34.31 ± 0.65 *^,B^	34.80 ± 0.81 ^B^	182.08 ± 0.92 ^B^	12.71 ± 0.50 ^B^	19.56 ± 0.76 ^B^	155.22 ± 3.15 *^,A^	438.68 ± 3.13 ^B^
**Job’s tear**									
Grain (polished)	Raw	6.67 ± 0.02 ^b^	-	-	40.62 ± 3.52 ^e^	-	-	21.07 ± 0.90 ^d^	61.69 ± 4.36 ^d^
	Boiled/Steamed	9.07 ± 0.10 *^,B^	-	-	61.84 ± 3.38 *^,D^	-	-	46.33 ± 2.49 *^,C^	108.16 ± 4.68 *^,D^
**Corn cereal**		0.55 ± 0.04	-	-	3.45 ± 0.27	-	-	49.52 ± 5.43	52.97 ± 5.66

^(1)^ Mean ± SD (*n* = 3). ^(2)^ * Means significantly differ between raw/dried and cooked within grain varieties using Student’s *t*-test (*p* < 0.05). ^(3) a–g^ Means with different lowercase letters in the same column significantly differ among raw/dried grain using Duncan’s multiple range test (*p* < 0.05). ^(4)^ - Not detected. ^(5) A–E^ Means with different uppercase letters in the same column significantly differ among cooked grain using Duncan’s multiple range test (*p* < 0.05). ^(6)^ 40 min for oat and barley; 10 min for Job’s tear; 4 min for buckwheat noodles.

**Table 6:** Phospholipid composition of wheat flour and its products.

		Lipid Contents (%)	Phospholipids (mg/100g of Freeze-Dried)				
			NAPE	PE	PC	LPC	Total PL
**Wheat flour**							
Pan frying powder		1.78 ± 0.08 ^(1),^*^,(2),c,(3)^	82.37 ± 15.96 ^a,(2)^	5.58 ± 1.55 ^b^	272.08 ± 40.28 ^ab^	63.05 ± 11.94 ^a^	423.08 ± 69.40 ^a^
Korean wheat flour		2.66 ± 0.08 ^a^	62.93 ± 1.33 ^a^	21.17 ± 0.29 ^a^	291.76 ± 13.27 ^a^	46.59 ± 1.13 ^b^	422.45 ± 15.02 ^a^
All-purpose wheat flour		2.09 ± 0.15 ^b^	63.14 ± 4.97 ^a^	- ^(4)^	239.92 ± 6.01 ^b^	49.08 ± 2.18 ^ab^	352.14 ± 12.75 ^a^
**Products**							
Ramen noodles	Dried	16.05 ± 0.12 *^,a^	63.19 ± 4.22 ^b^	-	140.23 ± 4.34 ^c^	103.64 ± 3.04 *^,(4),a^	307.06 ± 1.12 *^,c^
	Boiled ^(6)^	13.56 ± 0.05 ^A,(5)^	61.08 ± 3.63 ^B^	-	140.67 ± 9.09 ^C^	85.05 ± 2.94 ^D^	286.80 ± 6.54 ^C^
Plain noodles	Dried	1.21 ± 0.08 ^d^	62.94 ± 5.87 ^b^	-	234.50 ± 6.65 *^,a^	104.77 ± 5.12 ^a^	411.32 ± 17.54 ^a^
	Boiled	1.52 ± 0.01 *^,C^	64.68 ± 4.08 ^B^	-	195.06 ± 6.80 ^A^	187.10 ± 5.87 *^,B^	463.56 ± 11.96 *^,B^
Udon noodles	Dried	4.05 ± 0.06 *^,b^	-	-	43.61 ± 1.66 ^e^	61.78 ± 1.55 ^c^	105.39 ± 3.02 ^e^
	Boiled	1.19 ± 0.03 ^D^	-	-	78.22 ± 6.22 *^,E^	119.98 ± 7.73 *^,C^	211.09 ± 14.20 *^,D^
Jjolmyeon	Dried	0.57 ± 0.01 ^e^	24.39 ± 0.94 ^c^	-	107.29 ± 6.92 ^d^	69.06 ± 3.35 ^b^	207.62 ± 10.64 ^d^
	Boiled	0.69 ± 0.03 *^,E^	27.41 ± 1.37 *^,C^	-	114.50 ± 7.42 ^D^	85.85 ± 5.19 *^,D^	235.74 ± 13.97 ^CD^
Chopped noodles	Dried	1.21 ± 0.04 ^d^	88.72 ± 2.00 ^a^	-	207.97 ± 14.63 ^b^	72.74 ± 4.12 ^b^	377.97 ± 20.77 ^b^
	Boiled	1.19 ± 0.08 ^D^	111.23 ± 7.11 *^,A^	-	181.52 ± 30.16 ^AB^	257.85 ± 41.11 *^,A^	570.78 ± 80.95 *^,A^
Spaghetti	Dried	1.89 ± 0.15 *^,c^	63.92 ± 1.67 ^b^	-	206.61 ± 4.20 *^,b^	105.35 ± 4.32 ^a^	387.35 ± 10.52 ^b^
	Boiled	1.69 ± 0.01 ^B^	62.10 ± 0.28 ^B^	-	167.41 ± 1.22 ^B^	176.78 ± 5.02 *^,B^	418.31 ± 5.63 *^,B^

^(1)^ Mean ± SD. (*n* = 3). ^(2)^ * Means significantly differ between dried and boiled within various wheat products using Student’s *t*-test (*p* < 0.05). ^(3) a–e^ Means with different lowercase letters in the same column significantly differ among wheat flour and dried wheat products using Duncan’s multiple range test (*p* < 0.05). ^(4)^ - Not detected. ^(5) A–E^ Means with different uppercase letters in the same column significantly differ among boiled wheat products using Duncan’s multiple range test (*p* < 0.05). ^(6)^ 4.5 min for ramen noodles; 3 min for plain noodles; 4 min for udon; 9 min for jjolmyeon; 6 min for chopped noodles; 8 min for spaghetti.

**Table 7:** Phospholipid composition of vegetables.

Vegetables		Lipid Contents (%)	Phospholipids (mg/100g of Freeze-Dried)						
			NAPE	PE	PC	PS	PI	LPC	Total PL
Soybean sprout	Raw	19.05 ± 0.05 ^(1)^	- ^(2)^	95.28 ± 2.91	119.15 ± 2.32	-	98.39 ± 1.09	-	312.82 ± 3.93
	Blanched (1 min)	19.38 ± 0.22 ^NS,(3)^	-	376.13 ± 16.74 *^,(4)^	518.47 ± 21.16 *	-	108.82 ± 1.28 *	-	1003.43 ± 36.97 *
Deodeok	Raw	2.91 ± 0.27 ^a,(5)^	10.54 ± 0.45 ^b^	54.32 ± 1.22 ^b^	76.63 ± 2.35 ^c^	-	25.00 ± 0.56 ^c^	-	166.49 ± 2.76 ^c^
	Blanched (2 min)	2.66 ± 0.19 ^a^	11.85 ± 0.53 ^a^	97.46 ± 6.26 ^a^	120.77 ± 4.73 ^a^	-	36.34 ± 0.82 ^a^	-	266.43 ± 10.25 ^a^
	Roasted (5 min)	1.95 ± 0.04 ^b^	11.19 ± 0.28 ^ab^	94.13 ± 3.17 ^a^	109.96 ± 0.57 ^b^	-	27.62 ± 1.93 ^b^	-	242.91 ± 1.61 ^b^
Balloon flower, root	Raw	2.25 ± 0.02 ^a^	9.16 ± 0.12 ^a^	108.15 ± 1.79 ^b^	136.46 ± 5.10 ^c^	-	39.62 ± 1.68 ^ab^	-	293.39 ± 8.25 ^c^
	Blanched (2 min)	2.02 ± 0.07 ^b^	5.89 ± 0.10 ^b^	162.24 ± 9.02 ^a^	206.72 ± 3.62 ^a^	-	40.71 ± 0.38 ^a^	-	415.56 ± 12.71 ^a^
	Roasted (7 min)	1.90 ± 0.01 ^c^	6.19 ± 0.24 ^b^	118.41 ± 4.90 ^b^	175.23 ± 3.01 ^b^	-	37.48 ± 0.73 ^b^	-	337.31 ± 7.46 ^b^
Bracken	Boiled/Dried (5 min)	5.47 ± 0.10 ^b^	24.41 ± 0.83	248.35 ± 33.14	687.78 ± 34.58	-	95.08 ± 6.64	-	1055.61 ± 73.05
	Boiled/Dried/Boiled (5 min)	6.46 ± 0.02 ^a^	31.22 ± 1.61 *	383.53 ± 33.10 *	994.05 ± 27.71 *	-	140.61 ± 6.73 *	-	1549.41 ± 66.81 *
Korean cabbage	Raw	2.40 ± 0.06 ^b^	-	163.05 ± 7.89 ^b^	105.88 ± 0.27 ^b^	-	50.77 ± 2.15 ^a^	-	319.70 ± 8.40 ^b^
	Boiled (2 min)	2.74 ± 0.03 ^a^	-	195.81 ± 15.58 ^a^	350.82 ± 22.37 ^a^	-	49.45 ± 2.76 ^a^	-	596.08 ± 40.46 ^a^
	Salted (2 h)	2.20 ± 0.02 ^c^	-	198.42 ± 5.04 ^a^	30.70 ± 1.54 ^c^	-	37.26 ± 1.90 ^b^	-	266.38 ± 7.42 ^c^
Cabbage	Raw	4.04 ± 0.07 ^a^	-	139.62 ± 3.62 ^b^	285.88 ± 3.52 ^c^	-	76.39 ± 1.18 ^a^	-	501.89 ± 0.45 ^b^
	Blanched (1 min)	3.60 ± 0.01 ^b^	-	153.82 ± 4.82 ^a^	377.73 ± 0.95 ^a^	-	65.98 ± 1.92 ^b^	-	597.53 ± 3.08 ^a^
	Steamed (3 min)	3.16 ± 0.08 ^c^	-	119.76 ± 4.87 ^c^	311.94 ± 4.99 ^b^	-	65.69 ± 4.50 ^b^	-	497.39 ± 13.27 ^b^
Onion	Raw	1.36 ± 0.02 ^b^	-	58.93 ± 3.15 ^a^	95.82 ± 2.60 ^b^	-	6.73 ± 0.44 ^b^	-	166.70 ± 6.48 ^c^
	Blanched (1 min)	1.43 ± 0.01 ^a^	-	55.17 ± 3.62 ^a^	128.99 ± 5.16 ^a^	-	12.09 ± 0.69 ^a^	-	215.58 ± 7.33 ^a^
	Roasted (5 min)	1.45 ± 0.03 ^a^	-	35.67 ± 0.46 ^b^	129.28 ± 1.41 ^a^	-	12.75 ± 0.80 ^a^	-	188.50 ± 0.80 ^b^
Carrot	Raw	2.27 ± 0.04 ^a^	-	121.99 ± 3.52 ^b^	235.73 ± 5.23 ^c^	-	63.01 ± 3.15 ^a^	-	420.72 ± 11.37 ^c^
	Blanched (1 min)	1.98 ± 0.02 ^c^	-	122.78 ± 8.78 ^b^	305.20 ± 29.91 ^b^	-	42.69 ± 2.58 ^b^	-	470.67 ± 41.24 ^bc^
	Steamed (3 min)	2.08 ± 0.03 ^b^	-	240.04 ± 16.44 ^a^	461.69 ± 39.18 ^a^	-	68.38 ± 6.12 ^a^	-	770.11 ± 61.60 ^a^
	Stir-fried (2 min)	1.65 ± 0.01 ^d^	-	130.01 ± 3.06 ^b^	324.25 ± 8.44 ^b^	-	44.29 ± 1.42 ^b^	-	498.55 ± 10.31 ^b^
	Juice	0.15 ± 0.01 ^e^	-	1.99 ± 0.20 ^c^	6.71 ± 0.24 ^d^	-	0.80 ± 0.02 ^c^	0.44 ± 0.01	9.94 ± 0.15 ^d^
**Mushroom**									
Shiitake Mushroom	Dried/Raw	2.78 ± 0.05	-	507.16 ± 10.54	541.58 ± 5.58 *	273.10 ± 3.28 *	32.23 ± 0.73	16.76 ± 0.37	1370.83 ± 8.46 *
	Dried/Boiled (2 min)	3.09 ± 0.04 *	-	520.24 ± 13.88 ^NS^	330.17 ± 3.52	139.42 ± 2.06	36.77 ± 1.12 *	19.82 ± 0.64 *	1046.42 ± 20.23
King oyster mushroom	Raw	4.12 ± 0.03 ^b^	-	1161.79 ± 92.85 ^b^	768.77 ± 48.71 ^b^	244.75 ± 25.23 ^a^	67.04 ± 1.61 ^b^	46.84 ± 0.63	2289.21 ± 167.81 ^b^
	Boiled (1.5 min)	4.44 ± 0.06 ^b^	-	1458.66 ± 6.48 ^a^	990.95 ± 16.78 ^a^	278.86 ± 7.70 ^a^	71.19 ± 1.03 ^b^	-	2799.66 ± 29.68 ^a^
	Roasted (2 min)	6.33 ± 0.59 ^a^	-	1472.60 ± 26.30 ^a^	723.93 ± 25.11 ^b^	172.87 ± 21.70 ^b^	99.12 ± 8.06 ^a^	-	2468.52 ± 62.46 ^b^

^(1)^ Mean ± SD (*n* = 3). ^(2)^ - Not detected. ^(3) NS^ Indicates no significant difference between raw and boiled within mushroom varieties using Student’s *t*-test (*p* < 0.05). ^(4)^ * Means significantly differ between raw and boiled within mushroom varieties using Student’s *t*-test (*p* < 0.05). ^(5) a–d^ Means with different lowercase letters in the same column significantly differ among each vegetable and each mushroom using Duncan’s multiple range test (*p* < 0.05).

**Table 8:** Phospholipid composition of fruits.

		Lipid Contents (%)	Phospholipids (mg/100g of Freeze-Dried)			
			PE	PC	PI	Total PLs
**Citrus fruits**						
Mandarin orange	Redhyang	1.49 ± 0.03 ^(1),ef,(2)^	178.58 ± 9.38 ^bc^	140.46 ± 5.34 ^a^	- ^(3)^	319.04 ± 14.71 ^b^
	Cheonhyehyang	1.73 ± 0.09 ^c^	226.39 ± 9.75 ^a^	143.54 ± 4.04 ^a^	18.14 ± 1.11 ^b^	388.07 ± 13.56 ^a^
	Hallabong	1.39 ± 0.03 ^f^	167.83 ± 2.85 ^c^	126.16 ± 2.26 ^b^	-	293.99 ± 5.09 ^c^
	Hwanggeumhyang	1.55 ± 03.01 ^de^	181.48 ± 3.80 ^bc^	127.61 ± 2.06 ^b^	-	309.10 ± 5.67 ^bc^
	Satsuma (Regular cultivar)	1.81 ± 0.02 ^bc^	147.95 ± 4.98 ^d^	96.70 ± 2.78 ^d^	-	244.66 ± 7.77 ^d^
	Satsuma (Early-maturing cultivar)	1.95 ± 0.03 ^b^	176.46 ± 0.63 ^bc^	125.48 ± 0.58 ^b^	-	301.94 ± 1.11 ^bc^
Kumquat		1.80 ± 0.05 ^c^	123.40 ± 3.91 ^e^	101.54 ± 4.74 ^d^	9.11 ± 0.39 ^d^	234.05 ± 8.21 ^d^
Lime		1.67 ± 0.02 ^cd^	180.74 ± 7.74 ^bc^	114.61 ± 8.02 ^c^	15.14 ± 1.07 ^c^	310.49 ± 6.77 ^bc^
Lemon		5.73 ± 0.21 ^a^	183.00 ± 15.05 ^bc^	101.80 ± 1.47 ^d^	26.25 ± 1.41 ^a^	311.05 ± 14.98 ^bc^
**Blueberry**	New Hanover	3.13 ± 0.04	24.80 ± 1.02	37.44 ± 4.43	-	62.24 ± 3.41
**Pear**						
	Singo	0.75 ± 0.04 ^a^	41.48 ± 2.20 ^b^	85.78 ± 1.07 ^a^	18.44 ± 1.27 ^a^	145.70 ± 4.17 ^a^
	Won hwang	0.77 ± 0.02 ^a^	45.98 ± 1.84 ^a^	77.58 ± 0.86 ^b^	16.76 ± 0.19 ^a^	140.32 ± 2.55 ^b^
	Juice	0.02 ± 0.00 ^b^	0.07 ± 0.00 ^c^	0.22 ± 0.00 ^c^	-	0.29 ± 0.01 ^c^
**Apple**						
	Busa	0.72 ± 0.02 ^b^	53.92 ± 4.65 ^a^	67.42 ± 3.34 ^a^	11.77 ± 1.48 ^a^	133.12 ± 7.35 ^a^
	Busa (with skin)	1.26 ± 0.05 ^a^	56.57 ± 1.38 ^a^	69.95 ± 1.10 ^a^	9.69 ± 0.32 ^b^	136.22 ± 1.37 ^a^
	Juice	0.02 ± 0.00 ^d^	0.90 ± 0.11 ^b^	1.06 ± 0.07 ^b^	0.18 ± 0.02 ^c^	2.03 ± 0.05 ^b^
	Jam	0.38 ± 0.05 ^c^	1.84 ± 0.41 ^b^	2.39 ± 0.14^b^	-	4.22 ± 0.52 ^b^

^(1)^ Mean ± SD. (*n* = 3). ^(2) a–e^ Means with different lowercase letters in the same column significantly differ among fruit varieties using Duncan’s multiple range test (*p* < 0.05). ^(3)^ -; Not detected.

## Data Availability

The original contributions presented in the study are included in the article. Further inquiries can be directed to the corresponding authors.
